# Molecular and Morphological Characterizations of *Echinococcus granulosus* from Human and Animal Isolates in Kashan, Markazi Province, Iran

**Published:** 2017

**Authors:** Mohsen ARBABI, Majid PIRESTANI, Mahdi DELAVARI, Hossein HOOSHYAR, Amir ABDOLI, Shahab SARVI

**Affiliations:** 1. Dept. of Medical Parasitology and Mycology, Faculty of Medicine, Kashan University of Medical Sciences, Kashan, Iran; 2. Dept. of Medical Parasitology and Entomology, Faculty of Medicine, Tarbiat Modares University, Tehran, Iran; 3. Dept. of Medical Parasitology and Entomology, Faculty of Medicine, Mazandaran University, Sari, Iran

**Keywords:** *Echinococcus granulosus*, Molecular phylogenetics, Morphological characterization, PCR-RFLP, Iran

## Abstract

**Background:**

One of the most important zoonotic helminths in the world is known as *Echinococcus granulosus*. Different strains of the *E. granulosus* have been described based on morphological and molecular characterizations, however, there is limited information regarding the characteristics of the phenotypes and genotypes of *E. granulosus* in Iran.

**Methods:**

The present study was prepared to evaluate the phenotypic and genotypic diversity of *E. granulosus* isolates collected from human, goat, sheep, and cattle based on 19 standard morphometric parameters and mitochondrial and nuclear genes (*CO1, ND1*, and *ITS1*) in Kashan, Markazi Province, Iran during 2013–2014.

**Results:**

The biometric analysis for the 19 characters revealed that the 19 morphometric values of cattle isolates were exceptionally higher than human, goat, and sheep isolates (*P*<0.05). Molecular analysis confirms the morphological findings. Phylogenic analysis of the *CO1*, *NAD1* and *ITS1* genes for all isolates, independent of the host, revealed that the common sheep strain (G1) is traveling among livestock in Kashan and the strains are highly adapted to goats, cattle, sheep, and humans.

**Conclusion:**

Both morphological and molecular results of this study indicated that the only genotype G1 of *E. granulosus* travels between humans and other intermediate hosts of this parasite in the area study.

## Introduction

One of the most prevalent, chronic, complicated and an ignored zoonotic disease, caused by the metacestode phase of tapeworm named *Echinococcus granulosus* is cystic hydatid disease (CHD). The agent of CHD is a worldwide geographical distribution with a great medical and veterinary importance. This tapeworm affects the main organs of humans and domestic animals such as the liver, lungs, and brain (1–3).

CHD has a significant impact on humans as well as slaughtered animal health, with an estimated up to 1.2 million people infected and over 2 billion dollars in annual economic losses from organ condemnation in the slaughter industry (4). In fact, this infection is the leading risk factor for the production of livestock as well as the growing of livestock (5). Poverty, as well as poor sanitation in the raising of livestock, requires essential interventions to eliminate infections and use effective control programs to break the transmission cycle (6). Similarly, in most of the Mediterranean countries and region, hydatidosis is a major public health problem in Iran. Most of the infected areas were located in the northeastern and western of the country, where raising sheep and other livestock farming is common (7). Furthermore, in different regions of Iran, the number of new surgical cases of hydatidosis diagnosed each year is estimated from a nearly 1.18 to 3/ 100000 population (8) and the annual economic loss incurred in all ruminants slaughtered was estimated to be the US $232.2 million (9).

*E. granulosus* have remarkable forms of phenotypes in that distinguishable based on morphological and biochemical features, but these methods have no capacity for identification, accurate diversity, and interspecies variation of this species (10–13). Generally, the advent of modern and more sensitive molecular techniques is applied to discriminating a complex intra-specific strain of *E. granulosus* (12) and showed that the diverse groups of genetic alternatives make up the genotype of *E. granulosus* (14). Until now, molecular tools such as sequence comparison of mitochondrial DNA are available, which easily identify diversity and DNA polymorphism using suitable genetic markers such as mtDNA (CO1, ND1) genes in both intermediate and definitive hosts (15–17). This approach could help clarify the epidemiological situation and suit control programs of disease in a certain area (3). Analyses of mitochondrial and nuclear genomes have been in an extensive range to categorize strains of *E. granulosus* into distinct genotypes. To date based on phenotypic characters and analysis of gene sequences, a high intra-specific diversity, as well as a complex consist of at least 10 (G1–G10) distinct strains or genotypes. Five species of *E. granulosus* have been reported worldwide and have introduced the new molecular phylogeny and taxonomy of the parasite (18, 19). These species and genotypes are *E. granulosus* sensu stricto (G1-3, sheep/buffalo strains), *E. equinus* (G4, horse strain), *E. ortleppi* (G5, cattle strain), *E. canadenrsis* (G6-7, camel-pig strain, G8, American cervid strain, G10 Fennoscandian cervid strain) (15, 18, 20–23). Recently molecular studies described a new specific genotype, named *E. felidis* found in lions (24).

This genetic characterization and the extensive different variants have been very notable in better comprehension and understanding of the life cycle pattern (25, 26) as well as other biological features of *E. granulosus*, such as sensitivity to chemotherapy and pathologic patterns (27,28). The complex of G1-3 is the most major genotype that is responsible for human infection for most of the global burden. Among these variants, the most common are G1 (sheep strain) (29), while limited events have been attributed to the camel strain (30, 31). Other genotypes are rare pathogenesis of human disease. The G4 genotype (horse stain) have not affected human (12). The phylogeny for *E. granulosus* was reconstructed due to the structures of the entire mitochondrial genome (22) as well as several nuclear markers (32, 33). Iran is one of the most widespread areas of unilocular hydatid cyst disease where numerous classes of intermediate hosts are frequently infected with *E. granulosus.* Currently, numerous molecular studies demonstrated several genotypes of *E. granulosus,* including complex G1-3 (sheep/buffalo strains) and G6 (camel strain) have been genotypically recognized from sheep, cattle, camel, as well as humans in different parts of Iran. These investigations clearly showed that the G1 genotype of *E. granulosus* is the major cause of hydatidosis in various regions of the country (11, 17, 34–43).

Identification of *E. granulosus* strains has transmitted in countless regions using diverse methods, including morphology and molecular genetics, which all of these methods have demonstrated that they are beneficial when used to accompany. Furthermore, concurrent use of phenotypic and genotypic characterization could prove more precise and dependable information regarding the nature of diversity within *E. granulosus* strains (44). For the design of control strategies and clarifying epidemiology and taxonomy of parasite in an endemic area, identification of *E. granulosus* variants using molecular and genetic methods is very important (21, 45, 46). Detection of genetic diversity of the larval stage and adult worms of *E. granulosus* for accurate understanding patterns, the transmission in endemic regions of Iran is important, where more than one species of intermediate host is present (43,47, 48).

The aim of the present study was to identify, both genetically as well as morphologically characteristics of *E. granulosus* isolates from humans, as well as different domestic animals (sheep, goats and cattle) from Kashan region, center of Iran using the partial sequence of rDNA (ITS1) and mtDNA (CO1 and ND1) genes. Such knowledge should allow a better comprehension of the molecular epidemiology and control of hydatidosis, as well as determining the nature and extent strains of Echinococcus in various regions of Iran.

## Materials and Methods

### Sampling design

During the period Apr 2013–Feb 2014, internal organs with unilocular hydatid cyst were composed of 15 patients who underwent surgery at the Shahid Beheshti Hospital and 33 sheep, 20 cattle, as well as 32 goats slaughtered at abattoirs in Kashan city, Iran. All of the patients that suffered from cystic echinococcosis were identified by histopathological examination (PAS staining) and imaging of the respective tissues. Samples of hydatid fluid aspirated from each hydatid cyst were collected, transported into a single tube and washed 3 times with sterile PBS (pH 7.2). Before fixing samples in 70% ethanol, the fluid cysts were controlled for the occurrence of protoscolices. The possibility of protoscolices was checked microscopically by using 0.1% aqueous eosin stain. All specimens were stored in 4 °C for future analysis.

Approval to this study was granted by Kashan University of Medical Sciences Ethical Committee (document 2731).

### Morphological analysis

Four hundred protoscolices from 100 cysts were isolated from liver and lung of human, sheep, cattle and goats and were analyzed for morphological characterization. The isolated protoscolices from each infected host were recognized by the diagnostic keys (49). Measurements of character were made on both small as well as large hooks per rostellum from all of the 400 protoscolices for each isolate. The total length (TL), total number of hooks (NH), blade length (BL), total width (TW), blade width (BW), handle width (HW), handle length (HL), as well as the distance between the blade and guard (BGD), were calculated by a calibrated ocular micrometer at magnifications of ×100 and ×400. The isolated protoscolices were transmitted into an individual plate, washed 3 times with PBS, and saved in 70% ethanol until molecular works.

The morphological differences between all studied hosts were analyzed with ANOVA and Student’s *t*-test using SPSS ver. 16 (SPSS Inc., Chicago, IL, USA).

### Molecular characterization

#### Genomic DNA extraction

For the genomic DNA extraction, the protoscolices were thoroughly rinsed numerous times with sterile phosphate buffer solution (PBS) (pH 7.2) to eliminate the ethanol prior to DNA extraction (50). Genomic DNA was extracted using Kit (Bioneer; Korea), according to manufacturer’s instruction with brief modifications. Roughly, 2 ml packed volume of protoscolices were mechanically ground in 200μl lysis buffer and 30μl proteinase K and incubated at 55 °C for 3 min. The purified DNA was eluted to a final volume of 30–50 μl in elution buffer (EL) and stored at −20 °C until molecular analysis. The concentration and quality of the DNA were determined using both spectrophotometric and gel electrophoresis methods.

### PCR-RFLP analysis

Fragments of ITS1, ND1, and CO1 genes were amplified from each isolates using primer pairs, previously described (15, 17). PCRs containing between 100 ng of DNA was performed using the following conditions: 1.25 U Taq DNA polymerase, 1×Taq DNA polymerase buffer (20 mM Tris–HCl, pH 8.4, and 30 mM KCl, 0.04 mM dNTP mix, 1.5 mM MgCl2) and 20 pmol of each primer in a final volume of 25 μl. The PCR condition: 5 min at 95 °C (initial denaturation), 35 cycles of 45 sec at 95 °C, 30 sec at 57.8 °C, 52 °C and 47 °C for ITS1, COX1 and ND1, respectively and 45 sec at 72 °C and finally, 10 min at 72 °C (final extension). For each set of PCRs, positive and negative (no-DNA) controls were included (51). The PCR products, with a 100 bp DNA ladder, were separated by electrophoresis on 1.5% agarose gels, observed, and analyzed under UV transillumination. *E. granulosus* DNA samples were evaluated by PCRRFLP of the genes coding for ND1, CO1, and ITS1, as previously described (Bowles and McManus, 1993) with slight modification. All PCR products were digested using restriction enzymes, including *HaeIII*, *RsaI*, *HpaII* for ITS1, ND1, and COX1 respectively, using buffer recommended by the manufacturer (Thermo Scientific) in a final 20μl volume. The restriction fragments were separated by electrophoresis on 3.5% agarose gel, and stained with ethidium bromide and visualized under UV transillumination.

### Genomic DNA sequencing and phylogenetic analysis

Phylogenetic assay was determined by analyzing genomic (rDNA, ITS1) and mitochondrial DNA (mtDNA ND1 and CO1). For this propose, 27 amplicons, representing each unique RFLP profile, were selected. Furthermore, the different RFLP-PCR products were purified using the AccuPrep® Gel Purification Kit (Bioneer; Korea) according to the manufacturer’s guidelines. The concentration of DNA was estimated by comparison with a DNA Marker (100bp) in 3.5% agarose gel. All resulting PCR products were sequenced by targeting genes (ND1, COX1, and ITS1) in both directions using the said primers by the ABIPRISMTM 3130 Genetic Analyzer automated sequencer (Applied Biosystems, USA). All sequences were compared with sequences of *E. granulosus* available in GenBank sequences of all regional species using the Chromas software (version 3.1). The nucleotide sequence analysis was done using the BLAST algorithms from the National Center for Biotechnology. Phylogenetic trees and progression analyses were constructed using Tamura 3- parameter option of the neighbor-joining model with MEGA6 software (52). *Taenia multicepes* (JX535576) were used as an out-group. The bootstrap scores were calculated for 2000 replicates.

## Results

### Morphological analysis

The results of the morphometric characterizations 400 protoscolices of *E. granulosus* from Kashan, Iran are presented in Table 1.

**Table 1:** Morphological indices of *Echinococcus granulosus* protoscolices belonging to the G1 genotype, according to the host species

**Host**	**NO protoscolices per cyst Character**	**Sheep 100**	**Goat 100**	**Cattle 100**	**Human 100**	**Statistical differences *P*-value**
Protoscolex	Length	166.44±24.8	152.75±34.13	190±28.08	166.78±30.74	<0.001
	Width	131.89±19.7	125.94±19.72	152.45±22.78	128.32±23.24	<0.001
Sucker	Length	55.53±10.93	54.53±12.45	64.5±10.47	56.65±8.97	<0.05
	Width	40.38±9.58	38.05±8.63	48.15±11.69	40.20±9.41	<0.005
	Total length	23.88±1.88	23.74±2.37	27.43±2.05	23.78±1.49	<0.001
	Total width	8.95±1.53	8.68±1.61	9.80±1.64	8.75±0.88	>0.05
	Blade length	11.92±1.02	11.78±1.60	13.80±1.55	11.60±1.31	<0.001
Large Hook	Handle length	6.74±0.97	6.59±0.84	7.41±0.56	6.72±0.68	<0.05
	Number hook	35.24±2.50	34.34±2	37.2±2.28	35.2±2.48	<0.005
	Blade width	8.54±1.41	8.33±0.91	9.55±1.06	8.42±2.32	<0.05
	Handle width	4.02±0.55	3.91±0.63	4.74±0.62	4±0.76	<0.001
	Blade/Guard Distance	9.77±1.32	9.6±1.73	10.35±3.7	9.78±0.92	>0.05
	Total length	19.17±1.69	18.86±1.94	21.05±1.99	19.15±2.62	<0.01
	Total width	7.18±1.87	6.93±1.87	8.54±2.12	7.07±1.37	<0.001
	Blade length	7.80±1.42	7.68±1.14	9.70±2.08	7.97±1.07	<0.001
Small Hook	Handle length	6.87±1.68	6.59±1.12	8.13±1.21	6.83±1.14	<0.005
	Blade width	6.71±1.43	6.55±1.08	8.28±1.35	6.89±2.24	<0.001
	Handle width	3.82±0.40	3.79±0.27	4.38±0.55	3.69±0.18	<0.001
	Blade/Guard Distance	9.74±1.39	9.37±1.76	10.46±2.05	9.72±1.06	>0.05

The arrangement of small and large hooks in humans, sheep, goat, and cattle isolates were similar. The results clearly showed a high degree of phenotypic variation between protoscolices of human, sheep, goat and cattle isolates in Kashan area.

According to the data, all 19 morphometric values detected from large and small hooks demonstrated of cattle isolates have significantly higher isolates than humans, sheep, and goats, other than the additional morphometric values of cattle isolate, which was suggestively higher than the other (*P*<0.05).

According to these results, the mean length and width of protoscolices were: human (166.78±30.74 μm, 128.32±23.24 μm), sheep (166.44±24.8 μm, 131.89±19.7 μm), and goats (152.75±34.13 μm, 125.94±19.72 μm), whereas cattle isolates were 190±28.08 μm and 152.45±22.78 μm, respectively (P<0.001 in all isolates).

### Molecular analysis

**Fig. 1:**
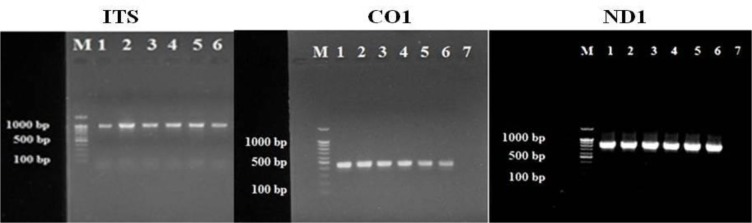
Electrophoresis analysis of ITS(1000 bp), CO1(444 bp) and ND1(830 bp). PCR amplification provided from sheep, goats, cattle and human *E. granolusus* samples (lane 1,2,: Sheep. 3: goats. 4: cattle. 5,6: Human) compared with the molecular weight marker(lane M, 100bp) and negative control (Lane 7)

The obtained samples were related to cysts of the liver (55%) and lung (45%). As predicted, PCR products size of a 1000 bp fragment of the ITS1 rDNA as well as 830 bp and 444 bp fragments of the ND1 and the CO1 mtDNA respectively were provided from all parasite samples (Fig. 1).

**Fig. 2:**
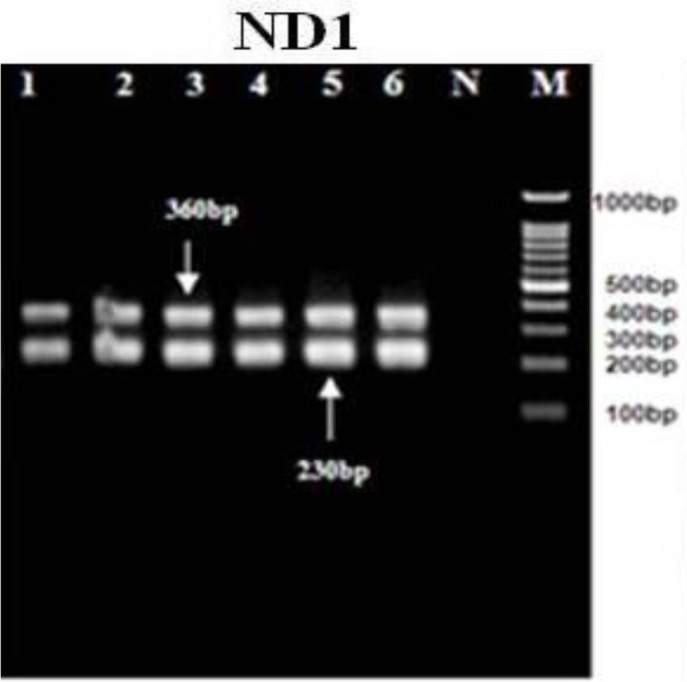
PCR-RFLP analyses of mitochondrial ND1 gene uses the restriction endonuclease *Rsa1*.1, 2: sheep. 3,4: goats. 5: cattle. 6: human. 7: Negative control (without DNA template). M: DNA ladder 100 bp

**Fig. 3:**
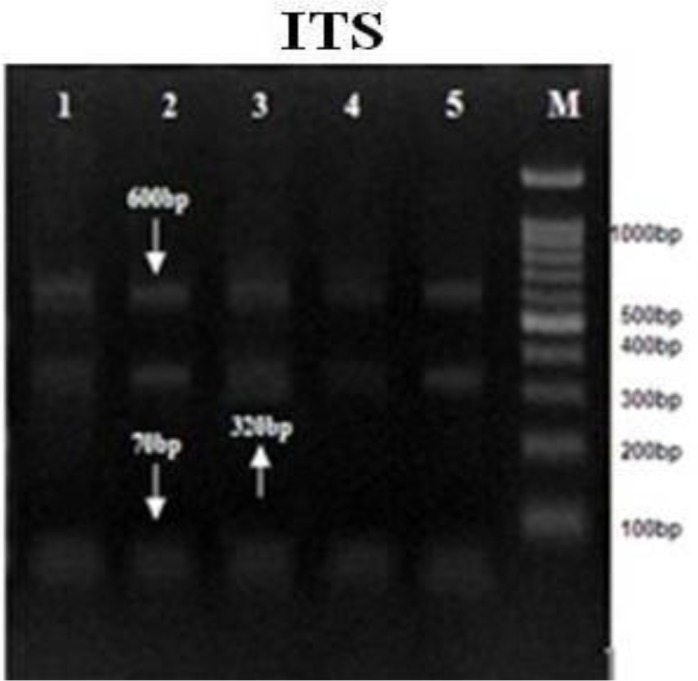
PCR-RFLP analyses of genomic ITS gene uses the restriction endonuclease *HaeIII.*1,2: sheep 3: goat. 4: cattle. 5: human. M : DNA ladder 100bp

**Fig. 4:**
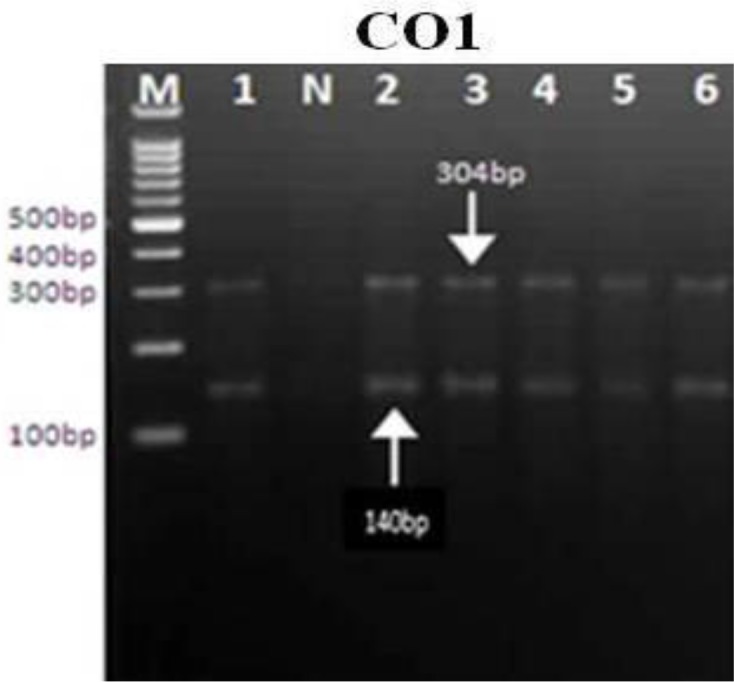
PCR-RFLP analyses of mitochondrial COX1 gene uses the restriction endonuclease *HpaII*.1,2: Sheep. 3,4: goats. 5: cattle. 6: human. M:DNA ladder 100 bp

All of the *E. granulosus* isolates were tested by the PCR-RFLP evaluation of the ITS1, ND1, and CO1 using restriction endonucleases (*HaeIII, RsaI*, and *HpaII*). The RFLP patterns of all cattle, sheep, goat, as well as human isolates, were identical (Fig. 2–4).

PCR-RFLP analysis using *RsaI* digestion demonstrated 2 bands of 230 and 360bp, *HaeIII* demonstrated 3 bands of 70, 320, and 600bp, and *HpaII* digestion demonstrated 2 bands of 304 and 140bp in all of the samples. Nine sequences from human, sheep, goats and cattle *E. granulosus* isolates have demonstrated and were submitted to the GenBank database with the accession nos.; KJ162552-KJ162560 for the ND1 gene, KJ162561 - KJ162569 for the CO1 gene and KJ363920-KJ363928 for the ITS gene. The evaluation of these two mitochondrial marker genes (ND1 and CO1) and 1 nuclear marker gene (ITS1) for the *E. granulosus* isolate confirmed the pretense of only the genotype G1 (common sheep strain) in all isolates.

**Fig. 5:**
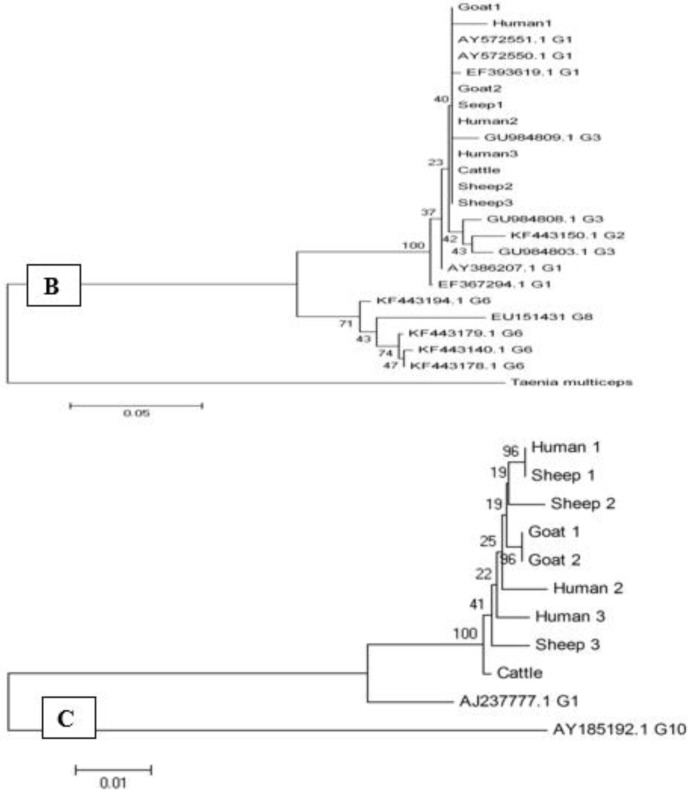
Phylogenetic relationships among *E. granolosus* inferred from nucleotide sequences of partial ND1 (A), CO1(B) and ITS(C) isolates from human, sheep, goat and cattle in Kashan, center Iran and other previous registered sequence of different areas. The evolutionary history was inferred using the neighbor-joining method. Number indicates bootstrap values (%) from 2000 replicates. The branches supported by 2000 bootstrap replicates

Analyzing of ND1 sequences showed similar point mutations at locations 123 (T and C), 157 (G and A), 296 (A and T), 300 (G and C), 306 (T and G) and 337 (T and C) for all the strains with G1 genotypes. Presence of point mutations at positions 97 (C to T), 201 (C to G), 265 (T to G), and 277 (A to G) was showed in the analyzed sequences of the ITS1 gene. The phylogenetic evaluation of the sequence data showed no host specificity among genotype. The phylogenetic analysis of concatenated sequences of CO1, ND1, and ITS1 was showed on the cluster, and represent all strains related to G1 genotype (Fig. 5).

## Discussion

Cystic echinococcosis is one of the essential public health complications in most of the Mediterranean region’s countries, including Iran, where the highest occurrence of disease in humans, as well as other intermediate hosts, is found (47, 48). Huge economic burdens and lack of resources are significant causes of the increasing of diseases as a public health problem (53).

In recent years, many investigations were undertaken regarding the importance of extending genotypic and phenotypic diversity in the dog tapeworm *E. granulosus,* the source of hydatid cyst disease (21). These variations have significant outcomes for the evolution of diagnostic techniques of *E. granulosus.* Molecular discrimination within and between *E. granulosus* detected by application of molecular methods triggered to divide this parasite to 10 distinct genotypes as each one occurs to have individual phenotypes characteristic (18). *E. granulosus* have 10 define genotypes (G1-10) in different regions of the world (18, 21). The most frequent and global public health problem associated with human disease is the common sheep strain (G1) distributed in all countries (5, 8, 18, 27, 28, 54). The design and control strategies are contingent upon such evidence and, in particular, mode of transmission is a threat to human health. *E. granulosus*, as a species, has extensive phenotypic and genotypic variation, which vary in morphology, life cycle patterns, host specificity, as well as other states (14).

For identification strains/genotypes of *E. granulosus*, according to extensive research, different methods have been carried out, and each of them have sensitivity and accuracy for detection of Iranian genotypes, however, using both morphological and molecular analytic methods could afford scientific and relevant knowledge around the extent and significance of dissimilarity within *E. granulosus*, the causative agent of hydatid disease (35, 54).

The present study showed a high level of morphological variations in large and small rostellar hook metacestodes of *E. granulosus* from cattle, sheep, goats, and humans. In spite of, biochemical analysis can prepare beneficial information on the recognition of genotypes of *E. granulosus* from various hosts, this approach can be undecided and limited about identify strains (14). Although morphological criteria for discrimination of *E. granulosus* may be doubtful, it is a quick, valid, and economical method for identifying *E. granulosus* strains in Iran, as well as a good tool for epidemiological studies (10, 55). Therefore, using both as molecular as well as morphological genetics methods simultaneously could provide more careful and dependable evidence regarding the nature and extent of variation within *E. granulosus* isolate (21).

In the present study, all 19 morphometric indices drastically among domestic animals (sheep, goat, and cattle) as well as human isolates obtained from the area study. Therefore, morphometric evaluation was discovered to be an advantageous tool for variance identifications of common strains of *E. granulosus* from Iran. Data acquired from the morphological analysis in the present study are comparable to those conveyed by other investigators of Iran (10, 35, 56). Analysis of the rostellar hooks of *E. granolusus* declared that in transmission of the larval stage to definitive hosts, characteristics of the hook may be stayed stable with less variation than adult worm. This could identify the strain of this tapeworm and the source of infection (49, 57). The total length of the large hook and the blade of large hook characters of Iranian animals were significantly different from Egyptian ones (40). These dissimilarities insinuate that there is possibly an inter-group heterogeneity among Egyptian as well as Iranian isolates. At present, various techniques have focused on the molecular identification of *E. granulosus*, while an increasing number of them used research on the parasites nuclear genes as well as mitochondrial NADH dehydrogenase 1(ND1) and cytochrome oxidase 1(CO1) (41–42). Recently in Iran, several molecular studies showed the diversity of mitochondrial and ribosomal genes and confirmed 3 distinct *E. granulosus* genotypes, including genotype G1 in cattle, sheep, camels, goats, as well as humans, genotype G3 in sheep, humans, buffalo, as well as cattle, and genotype G6 in humans, sheep, as well as camels in different geographic areas (12, 20, 26, 43–46). Camels, as well as sheep strains, are comparable identified in humans as well as cattle.

The results of this study showed that G1 genotype (sheep strain) was the predominant genotype of *E. granulosus* in infected humans as well as domestic animals, which is in agreement with other reports from Iran and other countries (17, 34, 36, 40, 48). The usage of mitochondrial DNA sequencing is primarily centered on its rapid evolution, emphasizing its significance in the biasness of closely affiliated organism as in the event of *Echinococcus* genotypes, which appear to be favorably homogenous evolutionary units (58).

## Conclusion

This study, for the first time based on morphometric and molecular-phylogenetic taxonomic, indicated that along strain (G1 sheep strain) of *E. granulosus* distributes amongst the intermediate masses of this parasite Kashan, Iran. Therefore, similar to additional transmittable diseases, hydatidosis is essential in being regarded as an important concern in the health policy makers’ decisions. Our genetic characterization of human and animal *E. granulosus* strain in Iran will be quite useful regarding determination strains of taxonomy and development prevention strategies as well as control programs of infected hosts, particularly when there is a shortage of evidence regarding the character of this worm that affects intermediate and definitive hosts. Further studies including additional sample sizes from diverse geographic regions of Iran are necessary for genetic mapping of *E. granulosus*.
